# Withanolide C Inhibits Proliferation of Breast Cancer Cells via Oxidative Stress-Mediated Apoptosis and DNA Damage

**DOI:** 10.3390/antiox9090873

**Published:** 2020-09-16

**Authors:** Tzu-Jung Yu, Jen-Yang Tang, Li-Ching Lin, Wan-Ju Lien, Yuan-Bin Cheng, Fang-Rong Chang, Fu Ou-Yang, Hsueh-Wei Chang

**Affiliations:** 1 Division of Breast Surgery and Department of Surgery, Kaohsiung Medical University Hospital, Kaohsiung 80708, Taiwan; u107500035@kmu.edu.tw; 2 Graduate Institute of Natural Products, Kaohsiung Medical University, Kaohsiung 80708, Taiwan; jmb@kmu.edu.tw (Y.-B.C.); aaronfrc@kmu.edu.tw (F.-R.C.); 3 Department of Radiation Oncology, Faculty of Medicine, College of Medicine, Kaohsiung Medical University, Kaohsiung 80708, Taiwan; reyata@kmu.edu.tw; 4 Department of Radiation Oncology, Kaohsiung Medical University Hospital, Kaohsiung 80708, Taiwan; 5 Department of Radiation Oncology, Chi-Mei Foundation Medical Center, Tainan 71004, Taiwan; 8508a6@mail.chimei.org.tw; 6 School of Medicine, Taipei Medical University, Taipei 11031, Taiwan; 7 Chung Hwa University Medical Technology, Tainan 71703, Taiwan; 8 Department of Biomedical Science and Environmental Biology, Ph.D Program in Life Sciences, College of Life Sciences, Kaohsiung Medical University, Kaohsiung 80708, Taiwan; u106023002@kmu.edu.tw; 9 Department of Marine Biotechnology and Resources, National Sun Yat-sen University, Kaohsiung 80424, Taiwan; 10 Center for Cancer Research, Kaohsiung Medical University, Kaohsiung 80708, Taiwan; 11 Cancer Center, Kaohsiung Medical University Hospital, Kaohsiung 80708, Taiwan; 12 Institute of Medical Science and Technology, National Sun Yat-sen University, Kaohsiung 80424, Taiwan

**Keywords:** withanolide, breast cancer, apoptosis, oxidative stress, DNA damage

## Abstract

Some withanolides, particularly the family of steroidal lactones, show anticancer effects, but this is rarely reported for withanolide C (WHC)—especially anti-breast cancer effects. The subject of this study is to evaluate the ability of WHC to regulate the proliferation of breast cancer cells, using both time and concentration in treatment with WHC. In terms of ATP depletion, WHC induced more antiproliferation to three breast cancer cell lines, SKBR3, MCF7, and MDA-MB-231, than to normal breast M10 cell lines. SKBR3 and MCF7 cells showing higher sensitivity to WHC were used to explore the antiproliferation mechanism. Flow cytometric apoptosis analyses showed that subG1 phase and annexin V population were increased in breast cancer cells after WHC treatment. Western blotting showed that cleaved forms of the apoptotic proteins poly (ADP-ribose) polymerase (c-PARP) and cleaved caspase 3 (c-Cas 3) were increased in breast cancer cells. Flow cytometric oxidative stress analyses showed that WHC triggered reactive oxygen species (ROS) and mitochondrial superoxide (MitoSOX) production as well as glutathione depletion. In contrast, normal breast M10 cells showed lower levels of ROS and annexin V expression than breast cancer cells. Flow cytometric DNA damage analyses showed that WHC triggered γH2AX and 8-oxo-2′-deoxyguanosine (8-oxodG) expression in breast cancer cells. Moreover, *N*-acetylcysteine (NAC) pretreatment reverted oxidative stress-mediated ATP depletion, apoptosis, and DNA damage. Therefore, WHC kills breast cancer cells depending on oxidative stress-associated mechanisms.

## 1. Introduction

Withanolide is a general name for at least 300 natural C-28 steroidal lactones [1,2]. Several kinds of withanolides are reported to have anticancer effects [3]. For example, withaferin A has anticancer effects on oral [4], colon [5], and breast [6] cancer cells. 4β-hydroxywithanolide [7] and withanone [8] caused selective killing against oral and breast cancer cells, respectively. Withanolide E had anticancer effects against renal cancer cells [9].

Accumulating evidence shows that a series of withanolides induce apoptosis of several cancer cells through oxidative stress. For example, withaferin A induces reactive oxygen species (ROS)-mediated apoptosis in oral [4], head and neck [10], and melanoma [11] cancer cells. Similarly, 4β-hydroxywithanolide induces ROS leading to apoptosis of oral cancer cells [7]. However, the mechanisms of anticancer action of other withanolides have not been fully explored as of yet. Further screening of other withanolide candidates for their antiproliferative potency is warranted.

Breast cancer is the most common cancer type in women worldwide. Breast cancer exhibits complex and heterogeneous characters with three typical clinical subtypes, such as hormone receptor (HR) positive, human epidermal growth factor receptor 2 (HER2) positive, and triple-negative breast cancer (TNBC). Breast cancer was also considered to have five molecular classification subtypes, including luminal A, luminal B, HER2, basal, and Claudin-low, with different combinations of positive and negative for HER2, HR, and estrogen receptor (ER) [12]. Different clinical subtypes of breast cancer show different responses to different treatments. Among these breast cancer subtypes, TNBC is the most difficult subtype for treatment due to the lack of HR, HER2, and ER overexpression. In addition to target therapy, chemotherapy provides an alternative mainstay for treatment of all subtypes of breast cancer cells. Clinical drugs were used for breast cancer therapy but they generally caused side effects to normal tissues. Therefore, new drug development for breast cancer therapy continues to be required.

Among the many withanolides, we noted that withanolide C (WHC), one of the active compounds isolated from the solanacean plant *Physalis peruviana*, was rarely investigated. Although WHC was shown to have anticancer potency in 2009, only IC_50_ values in a MTT study of liver (HepG2 and Hep3B), lung (A549) and breast (MDA-MB-231 and MCF7) cancer cells [13] were investigated as yet. The detailed anticancer mechanisms and antiproliferation effect of WHC on other cancer cells have not been yet explored. Therefore, the aim of the present study was to investigate its antiproliferation function and the mechanisms involved in WHC-treated breast cancer cell responses.

## 2. Materials and Methods

### 2.1. Cell Culture

SKBR3, MCF7, and MDA-MB-231 were the three cell lines for human breast cancer, obtained from the American Tissue Culture Collection (ATCC, Manassas, VA, USA). M10 is a normal human breast cell line, obtained from the Bioresource Collection and Research Center (BCRC; HsinChu, Taiwan). SKBR3, MCF7, and MDA-MB-231 cells are HER2+, luminal A, and Claudin-low subtypes of breast cancer cell lines exhibiting metastasis ability, respectively [14,15]. Dulbecco’s Modified Eagle Medium (DMEM)/F12 (3:2 mixture) with 10% bovine serum (Gibco, Grand Island, NE, USA), antibiotics, and glutamine were used for culturing the SKBR3, MCF7, and MDA-MB-231 cells. Alpha medium with 10% fetal bovine serum (Gibco) and common antibiotics (penicillin and streptomycin) were used for the M10 cells. The cells were cultured in a humidified atmosphere 5% CO_2_ at 37 °C.

### 2.2. ATP and MTS Assays

Double checking of the proliferation status was performed by both ATP and MTS assays. The cell viability in terms of cellular ATP content was measured by ATP-lite assay kit (PerkinElmer Life Sciences, Boston, MA, USA) [16]. The cell viability in terms of mitochondrial metabolic activity was measured by a colorimetric MTS assay (Promega Corporation, Madison, WI, USA) [17].

### 2.3. Drug Information

This purification of the WHC was performed as follows. The plant materials of *Physalis peruviana* were collected in Tainan county, in September 2017. The species was identified by Dr. Yuan-Bin Cheng and a voucher specimen (PPR-18) was deposited in the Graduate Institute of Natural Products, Kaohsiung Medical University. The air-dried roots of *P. peruviana* (20.0 kg) were extracted with MeOH (15 L) thrice to yield a crude extract. This extract was partitioned between water and EtOAc to get the EtOAc portion (45.2 g). The later portion was further partitioned between hexanes and 75% MeOH_aq_ to gain a terpene-enriched portion (26.8 g). This portion was subjected to a silica gel flash column stepwise eluting with hexanes/EtOAc/MeOH to furnish eight fractions. Fraction 5 (20.3 g) was separated by another silica gel column stepwise elution with methylene chloride and MeOH to provide six subfractions. Subfraction 5-3 (9.0 g) was purified by reverse phase column stepwise elution with MeOH and H_2_O to yield eight fractions. Fraction 5-3-4 (3.4 g) was isolated by silica gel open column stepwise elution with hexane and acetone to give a subfraction 5-3-4-1 (587.3 mg). Subfraction 5-3-4-1 was purified by reversed phase high performance liquid chromatography (RP-HPLC) (C_18_ column, 62% MeOH, isocratic) to produce WHC (40.0 mg).

*N*-acetylcysteine (NAC) (Sigma-Aldrich, St. Louis, MO, USA) was used as an inhibitor for oxidative stress [18,19]. The pretreatment condition for the NAC used in the cells was 10 mM for 1 h. Cisplatin (Selleckchem, Houston, TX, USA) was used as a positive control for treatment to breast cancer cells and normal breast cells. WHC, NAC, and cisplatin were dissolved in dimethyl sulfoxide (DMSO), double-distilled water, and phosphate-buffered saline (PBS) for drug treatments. For the control treatment, cells were cultured with a low concentration of a DMSO-containing medium, where all treatments (control, NAC, WHC, and NAC/WHC) had the same DMSO concentrations in the same experiments, as indicated.

### 2.4. Antibody Information

For Western blotting, the primary antibodies were specifically recognized of the cleaved forms of poly (ADP-ribose) polymerase (c-PARP) (Asp214) (D64E10), caspase 3 (c-Cas 3) (Asp175) (5A1E) (1:1000 dilution), and mAb-β-actin (control) (1:5000 dilution), which were obtained from Cell Signalling Technology Inc. (Danvers, MA, USA) and Sigma-Aldrich [20]. For flow cytometry, the primary antibodies for p-Histone H2A.X (Ser 139) (γH2AX) and the 8-OHdG antibody (E-8) Fluorescein isothiocyanate (FITC), as well as the Alexa 488-conjugated secondary antibody for the γH2AX primary antibody, were obtained from the Santa Cruz Biotechnology (Santa Cruz, CA, USA) and Cell Signalling Technology Inc.

### 2.5. Cell Cycle Assay

Cellular DNA contents were stained with 7-aminoactinomycin D (7AAD) (Biotium Inc., Hayward, CA, USA) (1 μg/mL, 30 min, 37 °C) as described previously [4]. A flow cytometer (Guava*^®^*easyCyte^TM^; Luminex, TX, USA) and FlowJo software (Becton-Dickinson; Franklin Lakes, NJ, USA) were used for cell cycle determination.

### 2.6. Annexin V/7AAD Assay

An Annexin V/7AAD dual staining kit (Strong Biotech Corp., Taipei, Taiwan) was chosen for apoptosis detection as previously described [17] and analyses were conducted using a BD Accuri C6 flow cytometer. Annexin V (10 μg/mL) and 7AAD (1 μg/mL) were used for dual staining at 37 °C for 30 min.

### 2.7. ROS and Glutathione (GSH) Assays

Cellular ROS was probed with 2′,7′-dichlorodihydrofluorescein diacetate (H_2_DCF-DA) (Sigma-Aldrich) (10 μM, 30 min, 37 °C) as previously described [21] and analyzed using a BD Accuri C6 flow cytometer. Cellular GSH was probed with CellTracker Green 5-chloromethylfluorescein (CMFDA) (Thermo Fisher Scientific, Carlsbad, CA, USA) (0.1 μM, 30 min, 37 °C) as previously described [22] and analyzed using a flow cytometer (Guava^®^ easyCyte^TM^) and FlowJo software (Becton-Dickinson).

### 2.8. Mitochondrial Superoxide (MitoSOX) Assay

MitoSOX was probed with MitoSOX™ Red (Thermo Fisher Scientific, Carlsbad, CA, USA) (50 nM, 30 min, 37 °C) and analyzed using a BD Accuri C6 flow cytometer as previously described [23].

### 2.9. γH2AX Assay

The primary antibody against γH2AX (1:500 dilution, 4 °C, 1 h) and its coupled Alexa 488-conjugated secondary antibody, as well as 7AAD (5 μg/mL, 30 min, 37 °C), were used [24]. An analysis was subsequently performed using a flow cytometer (Guava*^®^*easyCyte^TM^) and FlowJo software (Becton-Dickinson) as previously described.

### 2.10. 8-Oxo-2′-Deoxyguanosine (8-oxodG) Assay

Cells were probed with the antibody 8-OHdG (E-8) FITC (1:10000 dilution, 4 °C, 1 h) to detect an oxidative DNA damage marker (8-oxodG) [25]. An analysis was subsequently performed using a BD Accuri C6 flow cytometer as previously described.

### 2.11. Statistics

Significance for multiple comparison was determined by analysis of variance (ANOVA) coupled with Tukey’s HSD Post-Hoc Tests using JMP^®^ 12 software. For the purposes of multiple comparisons, data columns were marked with small letters. When data columns were without overlapping letters, there were significant differences between them.

## 3. Results

### 3.1. WHC Inhibits Proliferation of Breast Cancer and Normal Cells Involving ROS

**Figure 1:**
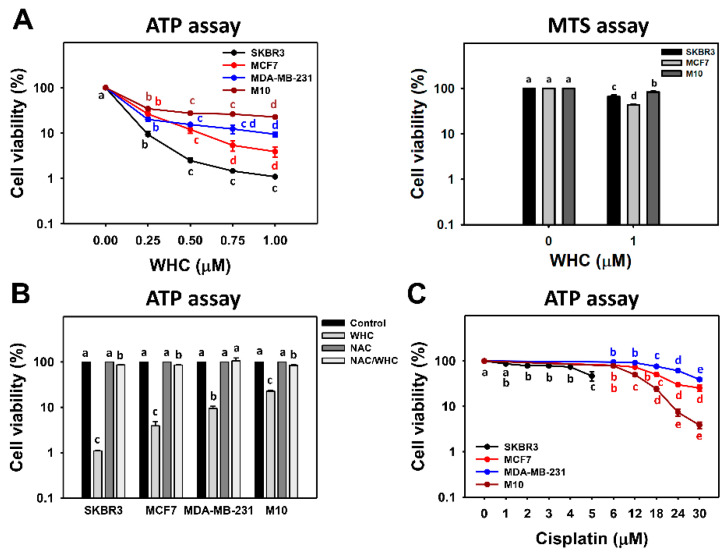
Withanolide C (WHC) inhibited the cell viabilities of breast cancer and normal breast cells differentially. Cell viabilities were determined by ATP assays. (**A**) ATP and MTS assays for determining cell viability after WHC treatment. For the ATP assay, breast cancer (SKBR3, MCF7 and MDA-MB-231) cells and breast normal (M10) cells were exposed to 0 (0.1% DMSO only), 0.25, 0.5, 0.75, and 1 μM of WHC for 48 h. For the MTS assay, breast cancer (SKBR3 and MCF7) cells and M10 cells were exposed to 0 (0.1% DMSO only) and 1 μM of WHC for 48 h. WHC also dissolved in the same concentration of DMSO. (**B**) *N*-acetylcysteine (NAC) pretreatment reversed WHC-induced ATP changes. Following pretreatment with NAC (10 mM for 1 h), cells were treated with the control and 1 μM of WHC for 48 h, i.e., NAC/WHC. (**C**) ATP assay for determining cell viability after cisplatin treatment for 48 h. Data are means ± SDs (*n* = 3). Results marked without overlapping letters show significant differences (*P* < 0.05 to 0.0001).

In terms of the ATP assay, at 48 h, WHC had decreased the cell viability of the three subtypes of breast cancer (SKBR3, MCF7 and MDA-MB-231) cells more than normal breast (M10) cells (Figure 1A, left). Accordingly, WHC exhibited higher antiproliferation of breast cancer cells than normal breast cells. For the sake of higher sensitivity to WHC, SKBR3 and MCF7 cells were chosen for other experiments to explore its antiproliferation mechanism in breast cancer cells. In terms of the MTS assay, at 48 h, WHC at 1 μM had decreased the cell viability of two subtypes of breast cancer (SKBR3 and MCF7) cells more than normal breast (M10) cells (Figure 1A, right), i.e., 66.6% and 43.0% vs. 83.4%, respectively.

There was a pretreatment with NAC to examine the effect of ROS on the antiproliferation function of WHC. The cell viabilities of breast cancer and normal cells after the WHC time course treatment were recovered to control by NAC pretreatment (Figure 1B). For comparison, the clinical drug cisplatin was used as a positive control to breast cancer cells and normal breast cells (Figure 1C). The drug sensitivity of WHC was higher than cisplatin for breast cancer cells. The cytotoxicity of WHC was lower than cisplatin for normal breast (M10) cells.

### 3.2. WHC Disturbs Cell Cycle Progression of Breast Cancer Cells

**Figure 2:**
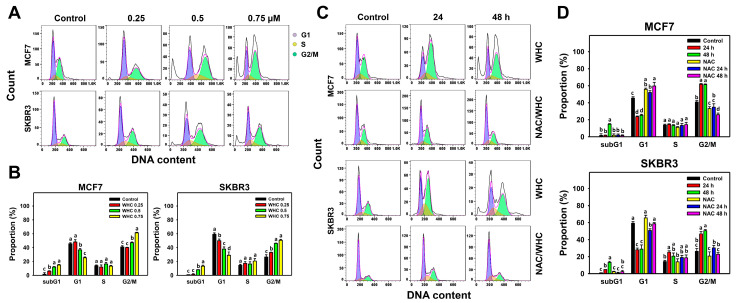
WHC disturbed cell cycle progression of breast cancer cells. (**A**,**B**) Cell cycle profiles and statistics for dose effects of WHC. Breast cancer (MCF7 and SKBR3) cells were treated with WHC (control (0.075% DMSO), 0.25, 0.5, and 0.75 μM, respectively) for 48 h. (**C**,**D**) NAC pretreatments reversed the WHC induced cell cycle disturbance. Following pretreatments with NAC (10 mM for 1 h), cells were treated with the control (0.075% DMSO) and 0.75 μM of WHC for 0, 24, and 48 h, i.e., NAC/WHC. Data are means ± SDs (*n* = 3). Results marked without overlapping letters show significant differences (*P* < 0.05 to 0.0001).

The dose and time course changes of cell cycle progression in breast cancer cells were determined by 7AAD flow cytometry (Figure 2A,C). WHC showed dose- and time-dependent increases in subG1 populations, decreases in G1 population, and increases in G2/M population in breast cancer (SKBR3 and MCF7) cells (Figure 2B,D).

NAC pretreatment was used to examine the effects of pm the WHC function of cell cycle disturbance. Cell cycle disturbance of breast cancer cells after the WHC time course treatment was recovered by NAC pretreatment (Figure 2D).

### 3.3. WHC Differentially Induces Apoptosis (Annexin V/7AAD) of Breast Cancer and Normal Cells

**Figure 3:**
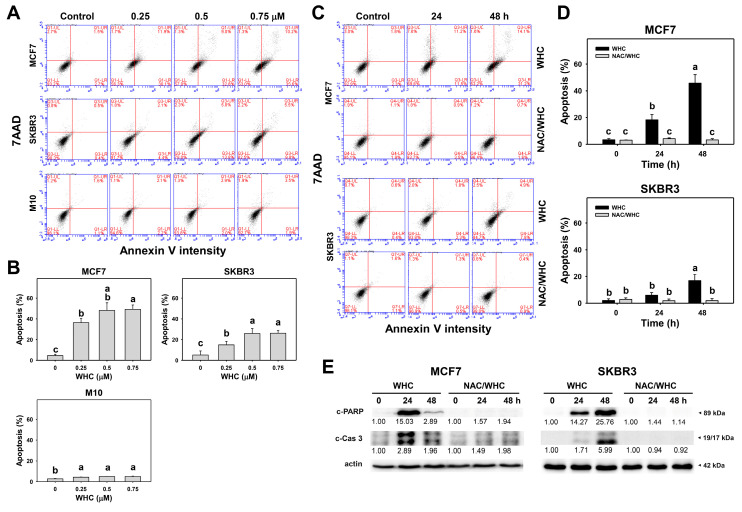
WHC induced apoptosis (annexin V/7AAD) differentially in breast cancer and normal breast cells. (**A**,**B**) Annexin V/7AAD profiles and statistics for dose effect of WHC. Breast cancer (MCF7 and SKBR3) cells and normal breast (M10) cells were treated with WHC (control (0.075% DMSO), 0.25, 0.5, and 0.75 μM) for 48 h. Annexin V (+) (%) was counted for the apoptosis (%). (**C**,**D**) NAC pretreatments reversed the WHC-induced apoptosis. Following pretreatments with NAC (10 mM for 1 h), cells were treated with control (0.075% DMSO) and 0.75 μM of WHC for 0, 24, and 48 h, i.e., NAC/WHC. Data are means ± SDs (*n* = 3). Results marked without overlapping letters showed significant differences (*P* < 0.05 to 0.001). (**E**) Apoptosis Western blotting of WHC. Cleaved PARP (c-PARP) and c-Cas 3 expressions were quantified referring to β-actin expression.

The dose and time course changes of annexin V/7AAD in breast cancer and normal breast cells were determined by flow cytometry (Figure 3A,C). The WHC treatment showed dose- and time-dependent increases in the apoptotic (annexin V) population of breast cancer (SKBR3 and MCF7) cells (Figure 3B,D), which was higher than that of normal breast (M10) cells (Figure 3B). The apoptosis changes were further confirmed by performing Western blotting. c-PAPR and c-Cas 3 were overexpressed in the breast cancer (SKBR3 and MCF7) cells (Figure 3E).

NAC pretreatment was used to examine the effects of ROS on apoptosis (annexin V) caused by WHC. Increasing apoptosis (annexin V) populations of breast cancer cells after the WHC time course treatment were recovered by NAC pretreatment (Figure 3D). Moreover, WHC-induced c-PAPR overexpression in breast cancer cells was suppressed by NAC pretreatment (Figure 3E). Similarly, WHC induced another apoptotic protein c-Cas 3 overexpression in breast cancer cells which was suppressed by NAC pretreatment.

### 3.4. WHC Differentially Induced ROS Generation and GSH Depletion of Breast Cancer and Normal Cells

**Figure 4:**
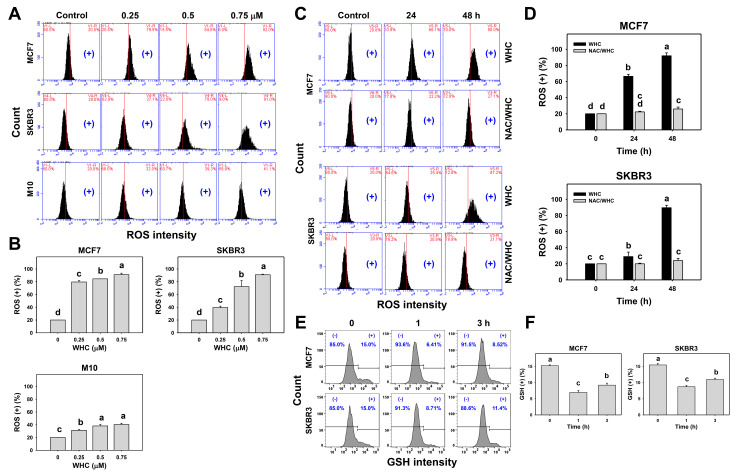
WHC differentially induced reactive oxygen species (ROS) generation among breast cancer and normal breast cells. (**A**,**B**) ROS profiles and statistics for dose effects to WHC. Breast cancer (MCF7 and SKBR3) cells and normal breast (M10) cells were treated with WHC (control (0.075% DMSO), 0.25 0.5, and 0.75 μM) for 48 h. (+) located at the right side of each profile is counted for ROS (+) (%). (**C**,**D**) NAC pretreatment reversed the WHC induced ROS generation. Following pretreatments with NAC (10 mM for 1 h), cells were treated with control (0.075% DMSO) and 0.75 μM of WHC for 0, 24, and 48 h, i.e., NAC/WHC. (**E**,**F**) GSH profiles and statistics for time course changes to WHC. Breast cancer MCF7 and SKBR3 cells were treated with WHC (control (0.075% DMSO) and 0.75 μM) for 0, 1, and 3 h. (+) located at right side of each profile is counted for GSH (+) (%). Data, means ± SDs (*n* = 3). Results marked without overlapping letters show significant differences (*P* < 0.05 to 0.0001).

The dose and time course changes of ROS generation in breast cancer and normal breast cells were determined by flow cytometry (Figure 4A,C). The WHC treatment showed dose- and time-dependent increases in the ROS (+) population in breast cancer (SKBR3 and MCF7) cells (Figure 4B,D), which was higher than that of normal breast (M10) cells (Figure 4B).

NAC pretreatment was used to examine the ROS function of WHC. Increasing ROS (+) populations of breast cancer cells after the WHC time course treatment were recovered by NAC pretreatment (Figure 4D). Moreover, the time course changes of the GSH content in breast cancer cells were determined by flow cytometry (Figure 4E). The WHC treatment showed decreases in the GSH (+) population in breast cancer (SKBR3 and MCF7) cells over time compared to the control (Figure 4F).

### 3.5. WHC Overexpresses MitoSOX in Breast Cancer Cells

**Figure 5:**
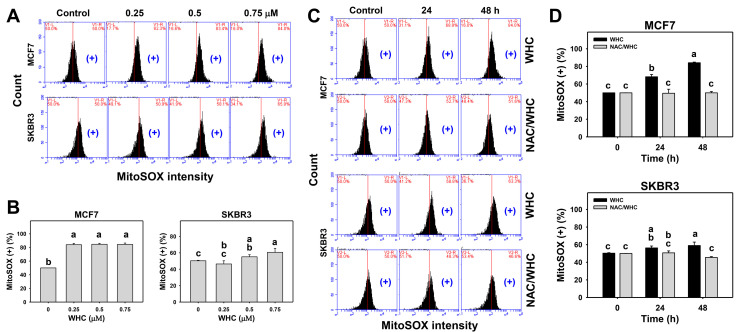
WHC differentially induces MitoSOX generation of breast cancer cells. (**A**,**B**) MitoSOX profiles and statistics for dose effects on WHC. Breast cancer MCF7 and SKBR3 cells and normal breast (M10) cells were treated with WHC (control (0.075% DMSO), 0.25, 0.5, and 0.75 μM) for 48 h. (+) located on the right side of each profile was counted for MitoSOX (+) (%). (**C**,**D**) NAC pretreatments reversed the WHC-induced MitoSOX generation. Following pretreatments with NAC (10 mM for 1 h), cells were treated with the control (0.075% DMSO) and 0.75 μM of WHC for 0, 24, and 48 h, i.e., NAC/WHC. Data are means ± SDs (*n* = 3). Results marked without overlapping letters show significant differences (*P* < 0.05 to 0.01).

The dose and time course changes of MitoSOX generation in breast cancer cells were determined by flow cytometry (Figure 5A,C). WHC showed dose- and time-dependent increases for MitoSOX (+) population in breast cancer (SKBR3 and MCF7) cells (Figure 5B,D).

NAC pretreatment was used to examine the effect of ROS on WHC-induced MitoSOX generation. Increasing MitoSOX (+) populations of breast cancer cells after the WHC time course treatment were recovered by NAC pretreatment (Figure 5D).

### 3.6. WHC Overexpresses γH2AX in Breast Cancer Cells

**Figure 6:**
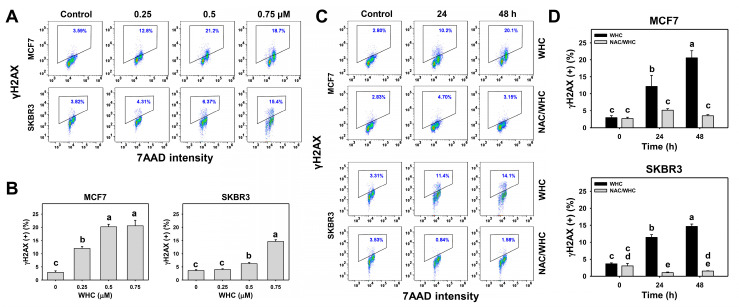
WHC differentially induced DNA double strand break (DSB) damage (γH2AX) of breast cancer cells. (**A**,**B**) γH2AX profiles and statistics for dose effects on WHC. Breast cancer (MCF7 and SKBR3) cells were treated with WHC (control (0.075% DMSO), 0.25, 0.5, and 0.75 μM) for 48 h. Box region of each profile is counted for γH2AX (+) (%). (**C**,**D**) NAC pretreatments reversed the WHC induced γH2AX generation. Following pretreatments with NAC (10 mM for 1 h), cells were treated with control (0.075% DMSO) and 0.75 μM of WHC for 0, 24, and 48 h, i.e., NAC/WHC. Data are means ± SDs (*n* = 3). Results marked without overlapping letters show significant differences (*P* < 0.005 to 0.0001).

The dose and time course changes of γH2AX expression in breast cancer cells were determined by flow cytometry (Figure 6A,C). WHC showed dose- and time-dependent increases in γH2AX (+) populations in breast cancer (SKBR3 and MCF7) cells (Figure 6B,D).

NAC pretreatment was used to examine the effect of ROS on WHC-induced γH2AX expression. Increasing γH2AX (+) populations of breast cancer cells after WHC time course treatment were recovered by NAC pretreatment (Figure 6D).

### 3.7. WHC Overexpresses 8-OxodG in Breast Cancer Cells

**Figure 7:**
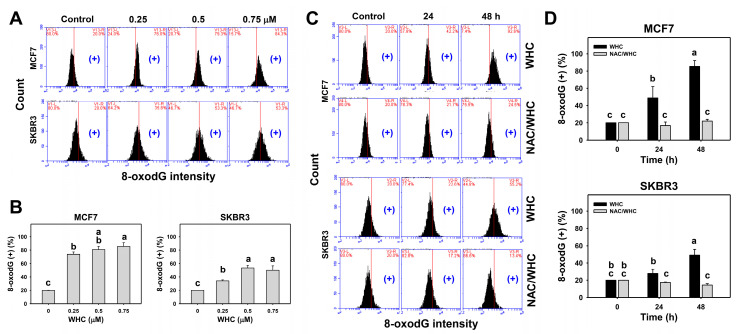
WHC differentially induced oxidative DNA damage (8-oxodG) of breast cancer cells. (**A**,**B**) 8-oxodG profiles and statistics for dose effects of WHC. Breast cancer (MCF7 and SKBR3) cells were treated with WHC (control (0.075% DMSO), 0.25, 0.5, and 0.75 μM) for 48 h. (+) located on the right side of each profile is counted for 8-oxodG (+) (%). (**C**,**D**) NAC pretreatments reversed the WHC induced 8-oxodG generation. Following pretreatments with NAC (10 mM for 1 h), cells were treated with control (0.075% DMSO) and 0.75 μM of WHC for 0, 24, and 48 h, i.e., NAC/WHC. Data are means ± SDs (*n* = 3). Results marked without overlapping letters show significant differences (*P* < 0.05 to 0.0001).

The dose and time course changes of 8-oxodG expression in breast cancer cells were determined by flow cytometry (Figure 7A,C). WHC treatment showed dose- and time-dependent increases in 8-oxodG (+) populations in breast cancer (SKBR3 and MCF7) cells (Figure 7B,D).

NAC pretreatment was used to examine the effect of ROS on WHC-induced 8-oxodG expression. Increasing 8-oxodG (+) populations of breast cancer cells after the WHC time course treatment were recovered by NAC pretreatment (Figure 7D).

## 4. Discussion

### 4.1. Withanolides Show Antiproliferative Potential for Cancer Cells

A number of withanolides showed antiproliferative activity against human breast cancer cell lines [26]. For example, the IC_50_ values for withanolide E (WHE) in a 72 h MTT study of breast cancer (MDA-MB-231 and MCF7) cells were 0.97 and 4.03 μM, respectively [13]. The IC_50_ values for withaferin A (WFA) after a 24 h MTS study of oral cancer (Ca9-22 and CAL 27) cells were 3 and 2 μM, respectively. However, WHC was not included in those study.

Recently, WHC cytotoxicity results were reported, i.e., the IC_50_ values for WHC in a 72 h MTT study of liver (HepG2 and Hep3B), lung (A549), and breast (MDA-MB-231 and MCF7) cancer cells were 0.13, 0.11, 1.24, 0.52, and 1.53 μM, respectively [13]. However, only cytotoxic IC_50_ values were reported, without addressing the detailed mechanisms.

In the current study, we found that the IC_50_ values for WHC in a 48 h ATP study of breast cancer (SKBR3, MCF7, and MDA-MB-231) cells and normal breast (M10) cells were 0.134, 0.172, 0.159, and 0.191 μM, respectively (Figure 1A, left). However, different kinds of assays are helpful for double checking cell proliferation. The proliferation statuses based on MTS and ATP assays were compared. In the 48 h MTS assay, normal breast cells (M10) showed higher viability than other breast cancer cells (MCF7 and SKBR3) (Figure 1A, right). The IC_50_ value for WHC in the 48 h MTS study of breast cancer (MCF7) cells was 0.89 μM. These results supported the concept that the luminescent ATP assay is more sensitive than the colorimetric MTS tetrazolium assay [27,28]. Moreover, this shows that WHC selectively kills breast cancer cells and is less harmful to normal breast cells. 

In comparison, the IC_50_ values for cisplatin in the 48 h MTS study of breast cancer (SKBR3 and MCF7) cells were 12.37 and 34.83 μM, respectively [22]. In the 48 h ATP study, the IC_50_ values for cisplatin for breast cancer cells (SKBR3, MCF7, and MDA-MB-231) and normal breast cells (M10) were 4.9, 17.9, 26.9, and 12.0 μM, respectively (Figure 1C). This shows that cisplatin non-selectively kills more normal breast (M10) cells than breast cancer (MCF7 and MDA-MB-231) cells. Therefore, WHC has a higher drug sensitivity and selectivity to antiproliferation of breast cancer cells than cisplatin. We further suggest using a xenograft assay of breast cancer cell lines to examine the anticancer effect of WHC in an animal model (e.g., zebrafish embryo, mouse) in vivo in the future.

### 4.2. WHC Exhibits Higher ATP Depletion for Breast Cancer Cells than Normal Breast Cells

ATP production is the main function of mitochondria. ATP depletion may occur coupled with MitoSOX production. For example, manoalide inhibits ATP production in 3D cultures and induces MitoSOX production [17]. Accordingly, ATP depletion reflects mitochondrial impairment. Moreover, mitochondrial damage induced by proteasome inhibition also overproduces MitoSOX, subsequently triggering cytosolic oxidation and leading to cell death [29]. Consistently, we found that WHC induced ATP depletion (Figure 1) and MitoSOX (Figure 5) and ROS generation (Figure 4) in breast cancer cells (SKBR3 and MCF7).

Since ATP depletion and MitoSOX generation are related to mitochondrial dysfunction, WHC may regulate other mitochondrial functions. For example, mitochondrial fitness [30] is regulated by mitochondrial dynamic changes, such as mitochondrial fusion and fission. Detailed investigation of the mitochondrial fitness of breast cancer cells after WHC treatments would further increase our understanding.

### 4.3. WHC Exhibiting Antioxidant Property May Contribute to Its Antiproliferation Ability to Breast Cancer Cells

Although some structures were not identified, such as WHC, 13 of 15 constituents of *Withania somnifera* root extract showed 2,2-Diphenyl-1-picrylhydrazyl (DPPH) scavenging effects, suggesting that most constituents of *W. somnifera* show an antioxidant ability [31]. Similarly, in our preliminary result, we found that WHC showed DPPH scavenging activity (Supplementary Figure S1), suggesting that WHC is an antioxidant agent. It is known that antioxidants perform dual functions for regulating cellular oxidative stress in a dose-dependent manner [32]. Under a high dose of an antioxidant, oxidative stress is induced. This concept may partly explain how WHC exhibiting an antioxidant property may induce oxidative stress which inhibits proliferation of breast cancer cells.

### 4.4. WHC Induces Differential Oxidative Stress and Apoptosis in Breast Cancer Cells

Generally, cancer cells show higher ROS levels than normal cells [33]. In chemotherapy, oxidative stress-modulating anticancer drugs commonly produce higher oxidative stress levels in cancer cells than in normal cells. Consequently, the drugs generate oxidative stress that is beyond ROS tolerance thresholds in cancer cells and inhibit cancer cell proliferation. In contrast, drug-generated oxidative stress in normal cells is less than their ROS tolerance threshold and is tolerated by normal cells. As a consequence, drug-generated oxidative stress selectively kills cancer cells [8,34,35,36,37]. This is partly attributed to the fact that oxidative stress can induce early apoptosis [38] by triggering mitochondrial dysfunction [39,40]. For example, withanone demonstrated selective killing and apoptosis against breast cancer cells [8]. Similarly, WHC induced more ATP depletion, ROS generation, and apoptosis (annexin V) in breast cancer cells (SKBR3 and MCF7) than in normal breast cells (Figure 1, Figure 3, and Figure 4), showing a selective killing potential to breast cancer cells.

Moreover, redox homeostasis is unbalanced when the antioxidant system is suppressed, resulting in oxidative stress overexpression after WHC treatment. This rational is further supported by our finding that GSH depletion was induced by WHC in breast cancer cells. Since oxidative stress is a kind of systemic cellular response, central metabolism/signaling pathways are likely involved here. For example, ROS may enhance glycolysis [41]. The TP53-inducible regulator of glycolysis and apoptosis (TIGAR) promotes the pentose phosphate pathway (PPP) [37] to generate the reduced form of nicotinamide adenine dinucleotide phosphate (NADPH) and prevent oxidative stress. Therefore, it warrants further investigation to explore the role of central metabolism/signaling pathways, such as glycolysis and PPP, in WHC-treated breast cancer cells in the future.

### 4.5. WHC Triggers DNA Damage in Breast Cancer Cells

Oxidative stress at a cytotoxic level is able to induce DNA damage [17,19,42,43]. Consistently, we found that WHC induced oxidative stress, such ROS and MitoSOX, and, therefore, was prone to lead to γH2AX DNA double strand breaks and 8-oxodG oxidative DNA damage in breast cancer cells (SKBR3 and MCF7) (Figure 6 and Figure 7).

### 4.6. NAC Reverts WHC-Induced Oxidative Stress Associated Changes in Breast Cancer Cells

All WHC-induced oxidative stress-associated changes were suppressed by NAC pretreatment. This holds for ATP depletion, cell cycle arrest, apoptosis, ROS/MitoSOX generation, and γH2AX/8-oxodG DNA damage. These results suggest that WHC-induced antiproliferation of breast cancer cells is oxidative stress-dependent.

## 5. Conclusions

A series of withanolides have been reported to exhibit antiproliferation potential against several types of cancer cells. However, the anticancer effect of WHC was rarely evaluated, especially on breast cancer cells. In the current study, the antiproliferation effect of WHC on breast cancer cells was confirmed. Detailed mechanisms of breast cancer cell antiproliferation were explored. The mechanisms were confirmed to depend on oxidative stress by NAC pretreatment experiments. Therefore, WHC showing antiproliferation effects represents a potential natural anticancer product against breast cancer cells by generating oxidative stress-mediated cell cycle changes, apoptosis, and DNA damage.

## Supplementary Materials

The following are available online at https://www.mdpi.com/2076-3921/9/9/873/s1, Figure S1: The DPPH radical scavenging activity of WHC.
