# Supplementary material for: Withanolide C Inhibits Proliferation of Breast Cancer Cells via Oxidative Stress-Mediated Apoptosis and DNA Damage
Source: Antioxidants (Basel). 2020 Sep 16;9(9):873. doi: 10.3390/antiox9090873 (PMC7555407; doi:10.3390/antiox9090873)
Supplement: Supplementary file 1 [file antioxidants-09-00873-s001.pdf]

antioxidants

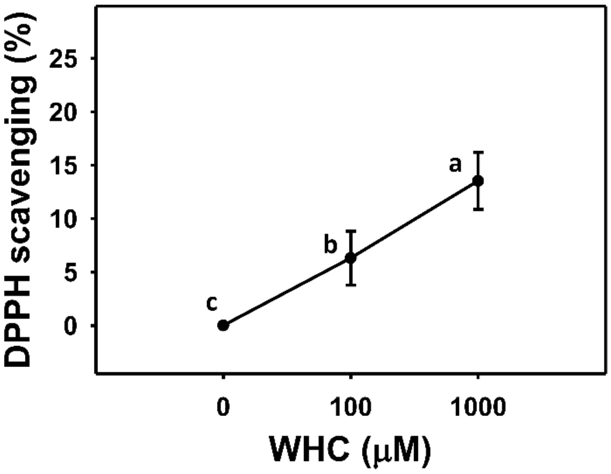

Supplementary Figure S1. The DPPH radical scavenging activity of WHC. WHC dose-responsively induces the DPPH, suggesting that WHC exhibits a potential antioxidant property. Significance for multiple comparison was determined by the analysis of variance (ANOVA) coupled with Tukey's HSD Post-Hoc Tests using JMP®12 software. Results marked without overlapping letters show significant differences ($P < 0.05$).
